# Satellite Laser-Ranging as a Probe of Fundamental Physics

**DOI:** 10.1038/s41598-019-52183-9

**Published:** 2019-11-04

**Authors:** Ignazio Ciufolini, Richard Matzner, Antonio Paolozzi, Erricos C. Pavlis, Giampiero Sindoni, John Ries, Vahe Gurzadyan, Rolf Koenig

**Affiliations:** 1 Dip. Ingegneria dell’Innovazione, Università del Salento, Lecce, and Centro Fermi, Rome, Italy; 2 Theory Center, University of Texas at Austin, Austin, USA; 3 Scuola di Ingegneria Aerospaziale, Sapienza Università di Roma, Roma, Italy; 4 Joint Center for Earth Systems Technology (JCET), University of Maryland, Baltimore County, USA; 5 Center for Space Research, University of Texas at Austin, Austin, USA; 6 Center for Cosmology and Astrophysics, Alikhanian National Laboratory and Yerevan State University, Yerevan, Armenia; 7 Helmholtz Centre Potsdam German Research Centre for Geosciences - GFZ, Potsdam, Germany

**Keywords:** Astronomy and planetary science, Space physics

## Abstract

Satellite laser-ranging is successfully used in space geodesy, geodynamics and Earth sciences; and to test fundamental physics and specific features of General Relativity. We present a confirmation to approximately one part in a billion of the fundamental weak equivalence principle (“uniqueness of free fall”) in the Earth’s gravitational field, obtained with three laser-ranged satellites, at previously untested range and with previously untested materials. The weak equivalence principle is at the foundation of General Relativity and of most gravitational theories.

## Introduction

**Figure 1:**
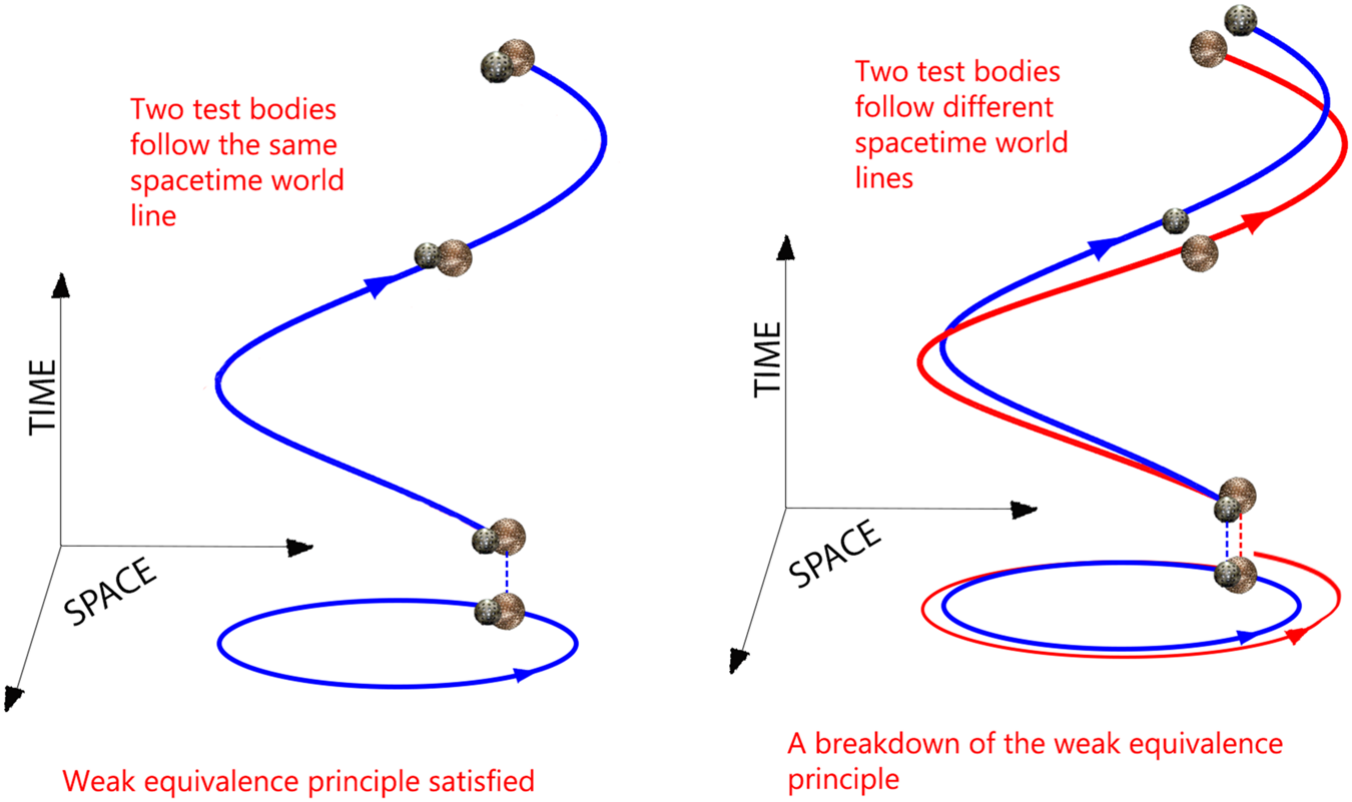
General Relativity and the Equivalence Principle. (**a**) Two bodies with the same initial conditions follow the same geodesic of spacetime. The projection of the spacetime geodesic onto a spatial plane is, for example, an ellipse (with suitable coordinates). Here, the third spatial dimension is suppressed and the much smaller relativistic precession of the pericenter is not shown. (**b**) If there is a violation of the uniqueness of free fall, two bodies with the same initial conditions will not follow the same spacetime curves and their projections onto a spatial plane will, for example, be two different ellipses.

General Relativity (GR) describes gravitational interaction via the geometry of spacetime whose dynamical curvature is determined by the distribution and motion of mass-energy; concurrently the motion of mass-energy is determined by the spacetime geometry. “Mass tells spacetime how to curve and spacetime tells mass how to move” (Wheeler^1^). However, for such a geometrical picture to work, any two particles, independently of their mass, composition and structure, must follow the same geometrical path of spacetime^2–4^. The weak equivalence principle states that the motion of any test particle due to the gravitational interaction with other bodies is independent of the mass, composition and structure of the particle. [A test particle is an electrically neutral particle, with negligible gravitational binding energy, negligible angular momentum and small enough that the inhomogeneities of the gravitational field within its volume have negligible effect on its motion.] Thus, the motion of planets, stars, and galaxies in the universe is simply dictated by the geometry of spacetime: they all follow purely geometrical curves of the spacetime called geodesic^1,2,5,6^. A geodesic is the generalization to a curved spacetime of a straight line of the flat Euclidean geometry. [The surface of a sphere is an example of a non-Euclidean geometry with positive curvature.] For example the motion of an artificial satellite around the Earth is not determined by the gravitational force that the Earth’s mass exerts on the satellite as in Newtonian theory. Rather the satellite is simply following a geometrical curve in spacetime, a geodesic, independent of its properties such as mass, composition, and structure, depending only on its initial conditions of position and velocity^5^. Then, for example, the observed (approximately) elliptical orbit of a satellite around the Earth is just the projection to our three dimensional space of the geodesic followed by the satellite in the four-dimensional curved spacetime geometry generated by the Earth’s mass (see Fig. 1a

).

There are a number of different formulations of the equivalence principle. The weak equivalence principle, also known as the Galilei equivalence principle, is based on the principle that the ratio of the inertial mass to the passive gravitational mass is the same for all bodies. This last formulation is also known as the Newton equivalence principle. The weak form is at the basis of most known viable theories of gravity. The medium form states that locally, in freely falling frames, all the non-gravitational laws of physics are the laws of special relativity^6^; the strong form includes gravitation itself in the local laws of physics, meaning that an external gravitational field cannot be detected in a freely falling frame by its influence on local gravitational phenomena. The medium form is at the basis of any gravitational theory based on a spacetime geometry described by a symmetric metric tensor, the so-called metric theories of gravitation, and the strong form is a cornerstone of GR. Since the weak equivalence principle underlies the geometrical structure of GR as well as our understanding of the dynamics of the universe and of astrophysical bodies, it has been tested in very accurate experiments^2–4^. Its tests go from the pendulum experiments (and inclined tables) of Galileo Galilei (about 1610), Christian Huygens (1673), Isaac Newton (1687) and Bessel (1832), to the classic torsion balance experiments of Eötvos^7^ (1889 and 1922) in the gravitational field of Earth (at a range from the center of ~6370 km). Roll, Krotkov and Dicke^8^ (1964) used aluminum and gold in the gravitational field of the Sun (at a range of ~1.5 · 10^8^ km) with a precision of ~10^−11^, and Braginsky and Panov^9^ (1972) used aluminum and platinum in the gravitational field of Sun with a precision ~10^−12^. The 2012 test by the University of Washington^10^ (the so-called “Eot-Wash” experiment) used a torsion balance to confirm the weak equivalence principle for beryllium-aluminum and beryllium-titanium test bodies in the field of the Earth to a precision of ~10^−13^. In April 2016 the space experiment *MICROSCOPE* of CNES (Centre National d’Études Spatial) was successfully inserted into orbit at an altitude of approximately 711 km. It was designed to test the equivalence principle to a precision of ~10^−15^ comparing the motion of two proof masses, one of titanium-aluminum alloy, and one of a platinum-rhodium alloy. The science phase of the mission lasted for about two years. First results (agreement with the weak equivalence principle to parts in 10^14^) were published in December 2017; the final measurements are expected this year (2019)^11^. Additional novel tests include one based on the time coincidence of gravitational radiation with electromagnetic observations from the LIGO event GW170817^12^. Also^13,14^, discuss the equivalence principle in the context of quantum systems.

Remarkably, GR even incorporates the strong equivalence principle^1^: gravitational energy (e.g. gravitational binding energy or the effective energy content of gravitational radiation) acts as a source (an active gravitational mass) for the gravitational field just like any other mass-energy, and responds to an external gravitational field (falls in that field) like any other passive gravitational mass. The strong equivalence principle has been validated by comparing acceleration of the Moon and the Earth toward the Sun using Lunar Laser Ranging, which measures the motion of the Moon relative to the Earth at the centimeter level. (The Earth’s fractional gravitational binding energy is about twenty times that of the Moon). Differential acceleration of the two bodies would lead to polarization of the Moon’s orbit; this has been excluded to parts in 10^13^ by Williams, Turyshev and Boggs^15^. Lunar Laser Ranging improvements of an order of magnitude to ~1 *mm* at the Apache Peak station will contribute to even better lunar equivalence principle results^16^. Archibald *et al*.^17^ studied the system PSR J0337 + 1715, and a gave a limit on the strong equivalence principle. The system consists of a triple: a tight (1.6 day) millisecond pulsar - white dwarf binary, in 327-day orbit about a distant white dwarf. Study shows the accelerations of the pulsar and its nearby white dwarf companion differ fractionally by no more than 2.6 × 10^−6^ as they fall toward the distant white dwarf.

Weak equivalence may be violated if there is a weak (of roughly the same strength as gravity) fundamental field that couples to matter differently from the universality of gravity. For instance, some theoretical constructs suggest an almost massless scalar field which couples to the nucleon number, rather than to the total mass-energy of the object. This scalar gravity would therefore be composition-dependent (thus violating the weak equivalence principle) since the fractional nuclear binding energy is different among elements. Different gravitational theories can exhibit a breakdown of the weak equivalence principle depending on the range, for example compared to the range of Yukawa-type deviations from the inverse square law of gravitation^18^ in a theory that couples to nucleon number. A composition dependent interaction between two bodies might be described by the following potential energy of a body 1 in the gravitational field of a body 2:

1 $$U(r)=-\,\frac{G{M}_{1}{M}_{2}}{r}(1+\frac{{b}_{1}{b}_{2}}{G{M}_{1}{M}_{2}}{e}^{-\frac{r}{\lambda }})$$

where −*GM*_1_*M*_2_/*r* is the standard Newtonian potential energy (representing the Newtonian gravitational theory as the lowest order approximation of GR), *G* is the gravitational constant, *M*_1_ and *M*_2_ are the masses of the two bodies, *b*_1_ and *b*_2_ are some composition dependent properties of bodies 1 and 2 defining the additional interaction, *r* is the distance between the two bodies and *λ* the Yukawa range of the interaction.

The ratio *b*/*M* will in general be different for each body and thus bodies with different compositions will fall with different acceleration, violating the uniqueness of free fall. Furthermore, a measurable deviation from the universality of free fall may depend not only on the material of the proof masses, nucleon number, etc., but also, as in Eq. (1), on the range of the experiment (an effective change of $G{M}_{\odot }$ with distance) unless *λ* ≈ ∞. Therefore it is important to test the equivalence principle with different materials and at different ranges; an important aspect of the present determination is the distance scale involved. In our analysis we assume $G{M}_{\odot }$ is a universal constant and cast the problem entirely in terms of the universality (or not) of the ratio of satellite inertial mass to passive gravitational mass.

## Test of Equivalence Principle: Laser-Ranged Satellites LARES, LAGEOS, and LAGEOS 2

We describe a test of the weak equivalence principle using for the first time freely falling high altitude laser-ranged satellites: LARES, made of sintered tungsten^19,20^; and LAGEOS^21^ and LAGEOS 2^22^, two almost identical satellites each composed of 57% aluminum shell/43% brass core by mass. These are materials never previously tested. Further details about the satellites are found in the Section Methods below. The number of well tracked dense laser ranged satellites is not large, so if new satellites meeting these criteria are launched their inclusion would improve our analysis to (at least partially) disentangle the weak equivalence result from a more controversial change of $G{M}_{\oplus }$ with distance. [Such a gradient could be the result of a violation of the crucial theorem that a gravitating sphere acts as a point mass (shell theorem). The most general form for the force to fulfill the shell theorem, *F*(*r*) = *Ar*^−2^ + Λ*r* contains^23^ a cosmological constant Λ, which LARES and LAGEOS data constrain. Constraints on modified gravity laws, including Yukawa type, are essential for GR’s Newtonian law as limit, as well as for understanding the dynamical features in the local group of galaxies and its vicinity (see, e.g.^24^)].

The self-gravities of all three satellites are negligible. By comparing the residual radial accelerations of these three satellites, we obtain a test validating the weak equivalence principle with an accuracy of ~10^−9^. The range of the test described here goes from ~7820 km from Earth’s center (altitude 1450 km) for the LARES satellite to ~12200 km from Earth’s center for LAGEOS and LAGEOS 2. Our test thus fills a distance gap not covered by the laboratory and Lunar Laser Ranging tests; any scale range in principle will constrain parameters entering the “fifth” force, phenomenology or coupled gravity models.

## Orbital Analysis and Results

We processed more than half a million normal points of the three satellites LARES, LAGEOS, and LAGEOS 2. The laser ranging normal points were processed using NASA’s orbital analysis and data reduction software GEODYN II^25^, and validated by the orbital modelers UTOPIA^26^, and EPOSOC^27^. The data analysis was based on the Earth gravity model GGM05S^28^. [The Earth gravity field model GGM05S was released in 2013, based on approximately 10 years of data of the GRACE (Gravity Recovery and Climate Experiment)^29,30^ spacecraft. It describes the Earth’s spherical harmonics up to degree 180. The NASA-DLR (Deutsche Zentrum für Luft- und Raumfahrt: the German Aerospace Center) GRACE space mission consists of twin spacecraft launched in a polar orbit at an altitude of approximately 400 km and ~200–250 km apart. The spacecraft range to each other using radar and are tracked by the global positioning satellites. GRACE has greatly improved our knowledge of the Earth’s gravitational field.] The GEODYN analysis includes Earth rotation from Global Navigation Satellite Systems (GNSS) and Very Long Baseline Interferometry (VLBI), Earth tides, solar radiation pressure, Earth albedo, thermal thrust, and lunar, solar and planetary perturbations. We analyzed the laser ranging data of the LARES, LAGEOS, and LAGEOS 2 satellites from February 2012 to December 2014. The laser ranging data for LARES, LAGEOS, and LAGEOS 2 were collected from more than 40 ILRS stations all over the world^31^.

If we include the acceleration due to the Earth’s quadrupole moment (the Earth’s oblateness measured by the *J*_2_ coefficent^32^), and the potential breakdown of the uniqueness of free fall, the radial acceleration *a*_*r*_ of an Earth satellite can be written:

2 $${a}_{r}=-\,\frac{{m}_{g}}{{m}_{i}}\frac{G{M}_{\oplus }}{{r}^{2}}[1-3{J}_{2}{(\frac{{R}_{\oplus }}{r})}^{2}{P}_{20}+\ldots ].$$

[Note that *a*_*r*_ is *not* the second time derivative $\ddot{r}$ of the radial coordinate *r*. Consider for instance circular motion where the radius *r* is constant, but ${a}_{r}=-\,r{\dot{\theta }}^{2}$]. Here, for simplicity, we have included within *m*_*g*_/*m*_*i*_ any breakdown of the uniqueness of free fall, for example of the type of the second term of Eq. (1). (*m*_*g*_ is the passive gravitational mass of the satellite and *m*_*i*_ its inertial mass; *m*_*g*_/*m*_*i*_ is is a universal constant in GR and Newtonian Physics, equal to unity by choice of units). ${M}_{\oplus }$ and ${R}_{\oplus }$ are the Earth’s mass and equatorial radius, *r* is the radial distance of the satellite from the Earth barycenter, and *P*_20_ is the associated Legendre function, of degree 2 and order 0, of the satellite latitude (see Methods). The product $G{M}_{\oplus }$ for the Earth is today measured^33^ to be 398600.4415 km^3^/sec^2^ (including the mass of the atmosphere) with an estimated *relative* (*one*-*sigma*) uncertainty of ~2 · 10^−9^. The Earth’s dimensionless quadrupole moment^34^ *J*_2_ is equal to 0.0010826358 with a *relative* uncertainty of ~10^−6^ to 10^−7^. According to the uniqueness of free fall, the ratio *m*_*g*_/*m*_*i*_ is the same for every test body. Here we consider the possibility that such a ratio may be different for aluminum/brass of the LAGEOS satellites, at a distance of ~12220 km from the Earth center, and for the tungsten alloy of the LARES satellite, at a distance of ~7820 km from the Earth’s center. On the basis of the LAGEOS, LAGEOS 2 and LARES laser-ranging observations, we then set an experimental limit on the deviation *δ*(*m*_*g*_/*m*_*i*_):

3 $$\delta (\frac{{m}_{g}}{{m}_{i}})={\frac{{m}_{g}}{{m}_{i}}|}_{tungsten}-{\frac{{m}_{g}}{{m}_{i}}|}_{alu{\min }um/brass}.$$

$\delta (G{M}_{\oplus })$, *δJ*_2_, *δ*(*m*_*g*_/*m*_*i*_) and the measurement error *δr* of the radial distance, *r*, of the three satellites are the main uncertainties in our estimation of the radial accelerations, Eq. (2), of the three satellites. See Eq. (9) below.

**Figure 2:**
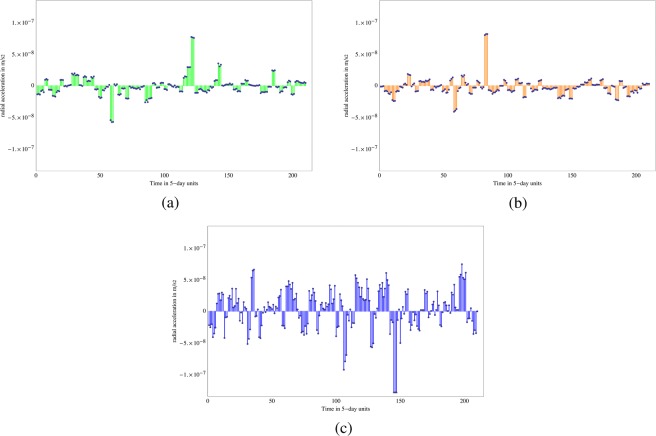
Residual radial accelerations. (**a**) Residual radial accelerations of LAGEOS. (**b**) Residual radial accelerations of LAGEOS 2. (**c**) Residual radial accelerations of LARES.

The radial accelerations of the three satellites are modeled with our orbital estimator GEODYN II^25^ using the nominal (fiducial) values of $G{M}_{\oplus }$, *J*_2_ and *m*_*g*_/*m*_*i*_ = 1 and the measured Earth-station to satellite distances. The observed-minus-modeled radial accelerations are computed for every five-day period. These residual radial accelerations of LAGEOS, LAGEOS 2 and LARES are shown respectively in Fig. 2a–c. The variations around the mean in these figures are due to the uncertainties in the deviations of the Earth’s gravity field from spherical symmetry, i.e. due to the uncertainties in higher Earth spherical harmonics. The residuals of LAGEOS shown in Fig. 2a are smaller than those of the other two satellites since the value of $G{M}_{\oplus }$ used in GEODYN was obtained^33^ using the LAGEOS laser-ranging data. We normalize *m*_*g*_/*m*_*i*_ = 1 for LAGEOS and the essentially identical (in both composition and altitude) LAGEOS 2. Thus *δ*(*m*_*g*_/*m*_*i*_) can only appear in consideration of LARES. Eqs (9) and (10) below give the relations between the variations of the accelerations, and the parameter variations. The long term average residual accelerations for LARES are comparable to those for LAGEOS 2 even though LARES orbits at a much lower altitude (Fig. 2c) and LARES undergoes larger single-point excursions.

We observe the average residual radial accelerations:

4 $$\begin{array}{rcl} < \delta {a}_{r}{ > }_{LAGEOS} & = & -4.056\cdot {10}^{-10}m/{s}^{2}\\  < \delta {a}_{r}{ > }_{LAGEOS2} & = & -2.217\cdot {10}^{-9}m/{s}^{2}\\  < \delta {a}_{r}{ > }_{LARES} & = & +2.834\cdot {10}^{-9}m/{s}^{2}\end{array}$$

here the angle brackets “< >” are long term averages.

The satellites’ residual radial accelerations are mainly due to the errors $\delta (G{M}_{\oplus })$, *δJ*_2_, *δ*(*m*_*g*_/*m*_*i*_) and the measurement error in the radial distance, δr, of the three satellites. Recent studies of the best station performance in ranging to the LAGEOS and LARES satellites suggest *one*-*sigma* $\delta r \sim 2\,mm$ for the LAGEOS satellites and $\delta r \sim 3\,mm$ for LARES, which we adopt.

The method is to take the three equations for the residual radial acceleration of each of the three satellites (e.g., in Eq. (10) in Methods for LARES). These can be viewed as giving a vector of radial accelerations (Eq. (4)) equal to a square matrix **M** times a vector of measurement uncertainties: (*δ*(*m*_*g*_/*m*_*i*_), $\delta (G{M}_{\oplus })$, *δJ*_2_), plus a term proportional to radial measurement uncertainty, + *other errors*. We invert this equation (multiply by **M**^−1^), which yields (Methods)):

$$(\begin{array}{c}\delta {({m}_{g}/{m}_{i})}_{LARES}\\ \delta (G{M}_{\oplus })/G{M}_{\oplus }\\ \delta J{}_{2}\end{array})=(\begin{array}{c}2.0\times {10}^{-10}\\ 7.3\times {10}^{-10}\\ 4.3\times {10}^{-9}\end{array})+(\begin{array}{c}\pm 1.1\times {10}^{-9}\\ \pm 2.9\times {10}^{-10}\\ \pm 3.0\times {10}^{-9}\end{array})$$

**Figure 3:**
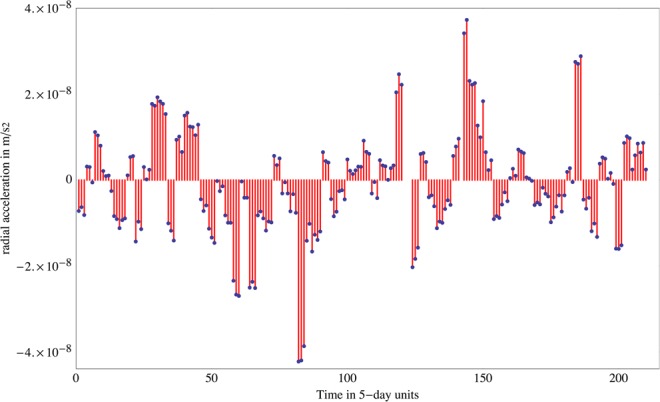
The residuals showing the deviation *δ*(*m*_*g*_/*m*_*i*_) for tungsten and aluminum/brass, Eq. (3), obtained by combining the residuals of the radial acceleration of the three satellites LARES, LAGEOS and LAGEOS 2. The variations over the mean are mainly due to the uncertainties in the variations of the Earth gravity field from spherical symmetry, i.e. to the uncertainties in the Earth spherical harmonics.

The column vector on the left represents the “decoupled” deviations of (*m*_*g*_/*m*_*i*_)_*LARES*_, $G{M}_{\oplus }$, and *J*_2_ from their nominal values. In particular *δ*(*m*_*g*_/*m*_*i*_) is independent of the uncertainties $\delta (G{M}_{\oplus })$ and *δ*(*J*_2_). The last column vector on the right represents the uncorrelated *δr* measurement errors; they turn out to dominate our result for *δ*(*m*_*g*_/*m*_*i*_). [Ranging to laser ranged satellites involves errors arising from atmospheric effects, photon statistics, and geometrical errors because the return comes from a retroreflector array which is not at the center of mass of the satellite.] Thus *δ*(*m*_*g*_/*m*_*i*_) is determined up to average residuals arising from random *δr* errors from the three satellites, and other smaller errors. See Methods. The resulting value of the deviation *δ*(*m*_*g*_/*m*_*i*_) for *tungsten* and *aluminum/brass*, Eq. (3), is:

5 $$\delta ({m}_{g}/{m}_{i})=2.0\times {10}^{-10}\pm 1.1\times {10}^{-9}$$

where ±1.1 · 10^−9^ is the estimated systematic error principally due to the error in the measurement of the radial distance. Uncertainties in the modeling of the radial accelerations due to the errors in the Earth’s spherical harmonics higher than the quadrupole moment, *J*_2_, and due to the errors in the modeling of atmospheric drag and of other non-gravitational perturbations, such as direct solar radiation pressure and Earth albedo, are included in the *other errors* above, and are much smaller. The combined residuals affecting *δ*(*m*_*g*_/*m*_*i*_) are shown in Fig. 3. Equation (5) shows a confirmation of the equivalence principle for the three satellites with an accuracy of ~±10^−9^.

## Discussion

Our test of the weak equivalence principle (uniqueness of free fall) using the laser ranged satellites LARES, LAGEOS and LAGEOS 2 fills a gap in the tests of this principle fundamental to Einstein’s gravitational theory of General Relativity.

Some alternative theories of gravitation predict deviations from the uniqueness of free fall that are enhanced at certain ranges depending on a typical scale length and are enhanced for different materials. Previous tests of the weak equivalence principle were Earth laboratory tests in the gravitational field of Earth (at a distance of ~6370 km from the center of the Earth) and at the MICROSCOPE distance of ~7000 km, and Earth laboratory tests in the gravitational field of the *Sun* (at ~1.5 × 10^8^ km). There were no tests at a range between 7820 km and 12270 km prior to the present test using the LAGEOS and LARES satellites. Furthermore the uniqueness of free fall was never previously confirmed comparing test bodies made of aluminum/brass and tungsten, such as the LAGEOS and LARES satellites. Our test has confirmed the validity of the weak equivalence principle for these metals over ranges 7820 km and 12270 km, to accuracy of one part per billion. Also, since LARES differs both in composition and in orbital radius from LAGEOS and LAGEOS 2 (which are very similar to one another in these properties) our observation can be viewed as constraining (*δG*)/*G*, again to ~ one part in 10^−9^, over the range 7820 to 12270 *km*.

## Methods

The orbits^19^ of the three satellites are almost circular, with very small orbital eccentricity. The LAGEOS satellite has semimajor axis 12270 km (altitude 5890 km), orbital eccentricity of 0.0045 and orbital inclination of 109.84°; LAGEOS 2 has semimajor axis 12160 km (altitude 5780 km), orbital eccentricity of 0.0135 and orbital inclination of 52.64°; LARES, semimajor axis 7820 km (altitude 1450 km), orbital eccentricity of 0.0008 and orbital inclination of 69.5°. In this analysis we assume all three satellites are in circular orbits. All three are passive, spherical, laser-ranged satellites. LAGEOS was launched in 1976 by NASA, and LAGEOS 2 in 1992 by ASI, the Italian Space Agency, and NASA. They are two almost identical spherical passive satellites covered with corner cube reflectors to reflect back laser pulses emitted by the stations of the satellite laser ranging (SLR) network of the International Laser Ranging Service (ILRS)^31^. SLR allows measurement of the radial position of the LAGEOS satellites with a median accuracy of the order two millimeters over an Earth-surface to satellite distance of ~6000 km. LARES is a satellite of ASI, launched in 2012 by ESA, the European Space Agency, with the new launch vehicle VEGA. LARES was designed to approach as closely as possible an ideal test particle^20^. This goal was achieved by minimizing the surface-to-mass ratio of the spherical satellite (the smallest of any artificial satellite), by reducing the number of its parts, by avoiding any protruding components, and by using a non-magnetic material. LARES carries laser retro-reflectors similar to those on LAGEOS and LAGEOS 2. Since LARES is at lower altitude, it is accessible to more ranging stations. Some of these stations have slightly reduced timing accuracy (compared to those that range to LAGEOS). As a result, the median accuracy of positioning of LARES is at the roughly the three millimeter level.

The classical gravitational potential of a spheroid, such as the Earth^32^, can be written:

6 $$U=\frac{G{M}_{\oplus }}{r}[1-{J}_{2}{(\frac{{R}_{\oplus }}{r})}^{2}{P}_{20}+\ldots ]$$

where ${M}_{\oplus }$ is the Earth’s mass, ${R}_{\oplus }$ its equatorial radius, *J*_2_ its quadrupole moment, *G* the gravitational constant, *r* is the radial distance from the origin and *P*_20_ is the Legendre associated function of degree 2 and order 0:

7 $${P}_{20}(sin{\varphi })=\frac{3}{2}si{n}^{2}{\varphi }-\frac{1}{2}$$

where *ϕ* is the latitude and in the expression (6) we have neglected higher order *P*_*n*0_ terms.

If there is a violation of the weak equivalence principle, the ratio *m*_*g*_/*m*_*i*_ of the gravitational mass to the inertial mass may be different between the aluminum/brass of the LAGEOS satellites and the tungsten alloy of the LARES satellite. Furthermore a composition dependent interaction between two bodies may depend on the distance between the two bodies and on the range of the interaction as described by Eq. (1). We have then indicated with *δ*(*m*_*g*_/*m*_*i*_) in Eq. (2) any breakdown of the uniqueness of free fall including, for example, one of the type of the second term of Eq. (1). The radial acceleration of a satellite, such as LAGEOS and LARES, can thus be written by Eq. (2) above. Therefore, the leading terms of the *residual, instantaneous* unmodeled radial accelerations of the LAGEOS and LARES satellites can be written:

8 $$\delta {a}_{r}\cong -\,\frac{G{M}_{\oplus }}{{r}^{2}}\,\delta (\frac{{m}_{g}}{{m}_{i}})-\frac{\delta (G{M}_{\oplus })}{{r}^{2}}+3\frac{G{M}_{\oplus }}{{r}^{2}}{(\frac{{R}_{\oplus }}{r})}^{2}{P}_{20}\,\delta {J}_{2}+2\frac{G{M}_{\oplus }}{{r}^{3}}\delta r$$

(one equation for each satellite).

We now set (*m*_*g*_/*m*_*i*_|_*aluminum*/*brass*_) ≡ 1 at the altitude of the LAGEOS satellites, but allow nonzero *δ*(*m*_*g*_/*m*_*i*_) for the sintered tungsten, lower orbiting LARES. Nonzero *δ*(*m*_*g*_/*m*_*i*_) indicates a violation of the equivalence principle between the LARES and the other satellites. Thus the *δ*(*m*_*g*_/*m*_*i*_) is possibly nonzero in Eq. (8) only for LARES.

In this formulation we assume a universal value of $G{M}_{\oplus }$ (the same value for all satellites). However we allow a possible offset $\delta (G{M}_{\oplus })$ between its observed, fiducial value, and its true value. (Hence $\delta (G{M}_{\oplus })$ is itself universal). Similarly, we assume a universal value of *J*_2_, with a possible offset *δJ*_2_ between its observed, fiducial value, and its true value; *δJ*_2_ is also universal.

The meaning of *δr* is different. It is the mean value of the uncertainty in the radial distance of each satellite from the Earth center of mass, mainly due to errors in the determination of the Earth center of mass, biases in laser ranges, errors in the modeling of the dispersion of the laser pulses by the troposphere, and uncertainties arising from determining the precise position of the retroreflector with respect to the center of mass of the satellite. Since these are mean values of uncorrelated errors, there are different values of *δr* for each satellite. We take 3 *mm* for LARES and 2 *mm* for the LAGEOS satellites.

With Eq. (8), we can write for LAGEOS (here angle brackets “< >” are *long term* averages):

9 $$\begin{array}{rcl} < \delta {a}_{r}{ > }_{LAGEOS} & \cong  & -\frac{\delta (G{M}_{\oplus })}{{r}_{LAGEOS}^{2}}+3\frac{G{M}_{\oplus }}{{r}_{LAGEOS}^{2}}{(\frac{{M}_{\oplus }}{{r}_{LAGEOS}})}^{2}\\  &  & \cdot \,(\frac{3}{4}si{n}^{2}{I}_{LAGEOS}-\frac{1}{2})\delta {J}_{2}+\frac{2G{M}_{\oplus }}{{r}_{LAGEOS}^{3}}\delta {r}_{LAGEOS}.\end{array}$$

The coefficient of *δJ*_2_ in Eq. (9) is the average over an orbit of the *P*_20_ Lagrange associated function of *sinϕ*, where *ϕ* is the latitude of the satellite. This function can be written as a function of the orbital inclination *I* and the true anomaly *f*:

$$sin{\varphi }=sinI\cdot sinf\mathrm{.}$$

Therefore the average value of *P*_20_ over one orbital period is:

$$ < {P}_{20} > =\frac{{\int }_{0}^{2\pi }\,(\frac{3}{2}{si}{{n}}^{2}I\cdot {si}{{n}}^{2}f-\frac{1}{2})df}{2\pi }=\frac{3}{4}{\sin }^{2}I-\frac{1}{2},$$

which is used in Eq. (9).

A similar expression to Eq. (9) holds for LAGEOS 2. However for LARES we take into account a possible deviation of *m*_*g*_/*m*_*i*_ from unity, so we have an additional term proportional to *δ*(*m*_*g*_/*m*_*i*_):

10 $$\begin{array}{rcl} < \delta {a}_{r}{ > }_{LARES} & \cong  & -\frac{\delta (\frac{{m}_{g}}{{m}_{i}})(G{M}_{\oplus })}{{r}_{LARES}^{2}}-\frac{\delta (G{M}_{\oplus })}{{r}_{LARES}^{2}}+3\frac{G{M}_{\oplus }}{{r}_{LARES}^{2}}{(\frac{{R}_{\oplus }}{{r}_{LARES}})}^{2}\\  &  & \cdot \,(\frac{3}{4}si{n}^{2}{I}_{LARES}-\frac{1}{2})\,\delta {J}_{2}+\frac{2G{M}_{\oplus }}{{r}_{LARES}^{3}}\delta {r}_{LARES}\end{array}$$

Start with Eq. (4) which gives an *observed* column matrix [<*δa*_*r*_>] of <*δa*_*r*_> values, (−4.056 × 10^−10^, −2.217 × 10^−9^, 2.834 × 10^−9^) *m*/*sec*^2^ for LAGEOS, LAGEOS 2, LARES, in that order. Work with normalized (fractional) quantities, so define a column matrix $\frac{ < \delta {a}_{r} > }{G{M}_{\oplus }/{r}^{2}}$, which normalizes each residual acceleration by the nominal acceleration at that radius. Rewrite the long time averages of Eqs (9) and (10) above in terms of the normalized residual accelerations:

11 $$\frac{ < \delta {a}_{r} > }{G{M}_{\oplus }/{r}^{2}}=-\,\delta (\frac{{m}_{g}}{{m}_{i}})-\frac{\delta (G{M}_{\oplus })}{G{M}_{\oplus }}+3{(\frac{{R}_{\oplus }}{r})}^{2} < {P}_{20} > \,\delta {J}_{2}+[2\frac{\delta r}{r}+other\,errors]\mathrm{.}$$

The *δr* and ALL the other errors from ranging are grouped into the last bracketed term. With the assumption that the $\frac{\delta r}{r}$ and *other* terms are uncorrelated, they should enter isotropically into this normalized equation.

Potential *other* error sources in the estimate of the anomalous radial accelerations include modeling errors in other gravitational perturbations, and in the non-gravitational perturbations. These error sources are much smaller than the one due to the quadrupole term *J*_2_^35^. Among the gravitational perturbations, there are the Earth harmonics, tidal, lunar, and planetary perturbations. The largest additional radial accelerations due to the Earth on the LAGEOS and LARES satellites are due to the harmonic *J*_4_. The contribution to the normalized residual radial acceleration due to *J*_4_ is: $5{(\frac{{R}_{\oplus }}{r})}^{4}{P}_{40}\,\delta {J}_{4}$. Here *P*_40_ is the associated Legendre function of degree 4 and order 0. The estimated^28^ uncertainty *δJ*_4_ is *δJ*_4_ ~ 2 × 10^−11 ^. This uncertainty is ~a factor of fifty below our ranging error. The periodicity of the various lunar and planetary perturbations averages their effect below the ranging error. The largest non-gravitational perturbation on the LAGEOS and LARES satellites, including the various radiation pressure perturbations and particle drag, is due to direct solar radiation pressure which produces mainly periodical effects. Further, its magnitude is $\sim {10}^{-6}\times$ the average radial acceleration due to *J*_2_ and the uncertainty in the modeling of the radiation pressure on LAGEOS is less than 1%^36^. Even if the direct solar radiation always acted to produce a constant radial acceleration, it and its uncertainty would be negligible with respect to the corresponding ones due to *J*_2_ and *δJ*_2_. (The non-gravitational perturbations are almost three times smaller on LARES than on LAGEOS, since the cross-sectional-area-to-mass ratio of LARES is about 2.7 times smaller than that of LAGEOS). A term not written in the dimensionless acceleration equation (Eq. (11)) would be proportional to *J*_2_ × *δI*; but the uncertainty in this term *J*_2_ × *δI* is much smaller than the ranging error (~10^−9^). [In solutions we find variances in inclination less than $0.1\,milliarcsec \sim 0.5\times {10}^{-9}(radians)$, but in Eq. (11) the inclination appears multiplied by ${J}_{2} \sim {10}^{-3}$, making the quantity *J*_2_ × *δI* much smaller than our ranging error.] Since these *other* terms, including those from variance in *I*, are very small compared to *δr*, they will be henceforth dropped.

Now, ignore for the moment the $[\frac{2\delta r}{r}]$ terms. Then Eq. (11) is of the form

12 $$[\frac{ < \delta {a}_{r} > }{G{M}_{\oplus }/{r}^{2}}]=[{\rm{M}}][fractional\,changes]$$

where $[\frac{ < \delta {a}_{r} > }{G{M}_{\oplus }/\,{r}^{2}}]$ is the column matrix of these quantities for LAGEOS, LAGEOS 2, and LARES; [*fractional changes*] is the column matrix [*δ*(*m*_*g*_/*m*_*i*_), $\frac{\delta (G{M}_{\oplus })}{G{M}_{\oplus }}$*, δJ*_20_]; and [M] is the matrix of coefficients.

We need to compute the orbit mean of *P*_20_ for each satellite, which is $\frac{1}{2}(\frac{3}{2}si{n}^{2}I-1)$ = 0.164, −0.026, 0.158, for LAGEOS, LAGEOS 2, and LARES respectively.

Also, ${({R}_{\oplus }/r)}^{2}=0.27$ for LAGEOS and LAGEOS 2, and 0.66 for LARES, so the coefficient of *δJ*_2_ in Eq. (11) is ${C}_{i}=3{(\frac{{R}_{\oplus }}{r})}^{2} < {P}_{20}\, > =0.1325,-0.0216,0.308$. These values correspond to *C*_1_ (LAGEOS), *C*_2_ (LAGEOS 2) and *C*_3_ (LARES). We will also need *C*_1_ − *C*_2_ = 0.1541, *C*_2_ − *C*_3_ = −0.3296, *C*_3_ − *C*_1_ = 0.1755.

As noted, we set *δ*(*m*_*g*_/*m*_*i*_) to zero for LAGEOS and LAGEOS 2. We also make the approximation that their radii are the same. Then the matrix in Eq. (12) is

$$[{\rm{M}}]=(\begin{array}{ccc}0 & -1 & {C}_{1}\\ 0 & -1 & {C}_{2}\\ -1 & -1 & {C}_{3}\end{array})$$

Its inverse is

$${[{\rm{M}}]}^{-1}=\frac{1}{{C}_{2}-{C}_{1}}(\begin{array}{ccc}{C}_{2}-{C}_{3} & {C}_{3}-{C}_{1} & {C}_{1}-{C}_{2}\\ -{C}_{2} & {C}_{1} & 0\\ -1 & 1 & 0\end{array})\mathrm{.}$$

Thus

$$(\begin{array}{c}\delta ({m}_{g}/{m}_{i})\\ \delta (G{M}_{\oplus })/G{M}_{\oplus }\\ \delta J\end{array})=\frac{1}{{C}_{2}-{C}_{1}}(\begin{array}{ccc}{C}_{2}-{C}_{3} & {C}_{3}-{C}_{1} & {C}_{1}-{C}_{2}\\ -{C}_{2} & {C}_{1} & 0\\ -1 & 1 & 0\end{array})\,[(\begin{array}{c}{[\frac{ < \delta {a}_{r} > }{G{M}_{\oplus }/{r}^{2}}]}_{1}\\ {[\frac{ < \delta {a}_{r} > }{G{M}_{\oplus }/{r}^{2}}]}_{2}\\ {[\frac{ < \delta {a}_{r} > }{G{M}_{\oplus }/{r}^{2}}]}_{3}\end{array})]$$

Define $G{M}_{\oplus }=398600\,k{m}^{3}/se{c}^{2}$ and express *δa*_*r*_ in *km*/*sec*^2^. Then we have the dimensionless quantities

$$(\begin{array}{c}{[\frac{ < \delta {a}_{r} > }{G{M}_{\oplus }/{r}^{2}}]}_{1}\\ {[\frac{ < \delta {a}_{r} > }{G{M}_{\oplus }/{r}^{2}}]}_{2}\\ {[\frac{ < \delta {a}_{r} > }{G{M}_{\oplus }/{r}^{2}}]}_{3}\end{array})=(\begin{array}{c}(\,-\,4.056\times {10}^{-13})/\mathrm{[398600}/{\mathrm{(12270)}}^{2}]\\ (\,-\,2.217\times {10}^{-12})/\mathrm{[398600}/{\mathrm{(12160)}}^{2}]\\ (\,+\,2.834\times {10}^{-12})/\mathrm{[398600}/{\mathrm{(7820)}}^{2}]\end{array})=(\begin{array}{c}-1.53\times {10}^{-10}\\ -8.22\times {10}^{-10}\\ +4.35\times {10}^{-10}\end{array})$$

Then inverting Eq. (12) (and setting the *δr* terms to zero) gives:

$$(\begin{array}{c}\delta ({m}_{g}/{m}_{i})\\ \delta (G{M}_{\oplus })/G{M}_{\oplus }\\ \delta J\end{array})=(\begin{array}{c}+2.0\times {10}^{-10}\\ +7.3\times {10}^{-10}\\ +4.3\times {10}^{-9}\end{array})$$

We now address the last term in Eq. (11), the column matrix of (2*δr*/*r*)_*i*_, which gives the magnitude of the uncorrelated errors for the satellites. These are the largest remaining uncontrolled errors.

Consider the 2*δr*/*r*-induced errors in the equivalence principle term:

13 $$\begin{array}{rcl}error(\delta ({m}_{g}/{m}_{i})) & = & -\frac{1}{{C}_{2}-{C}_{1}}[({C}_{2}-{C}_{3}){(2\delta r/r)}_{1} \mbox{+} +\mbox{''}({C}_{3}-{C}_{1}){(2\delta r/r)}_{2}\\  &  &  \mbox{+} +\mbox{''}({C}_{1}-{C}_{2}){(2\delta r/r)}_{3}]\end{array}$$

We use the quotes on the operators “+” because in fact the error *δr* is uncorrelated between satellites, so we add these errors by quadrature. Also, though this term has an explicit “−” sign, it is actually stochastic, so contributes “±” to the errors.

Thus the full statement of our result is:

$$(\begin{array}{c}\delta ({m}_{g}/{m}_{i})\\ \delta (G{M}_{\oplus })/G{M}_{\oplus }\\ \delta J\end{array})=(\begin{array}{c}+2.0\times {10}^{-10}\\ +7.3\times {10}^{-10}\\ +4.3\times {10}^{-9}\end{array})+(\begin{array}{c}\pm 1.1\times {10}^{-9}\\ \pm 2.9\times {10}^{-10}\\ \pm 3.0\times {10}^{-9}\end{array})$$

The physical result from these calculations is our statement of the equivalence principle: *δ*(*m*_*g*_/*m*_*i*_) = 2.0 × 10^−10^ ± 1.1 × 10^−9^ among the three satellites, consistent with the result *zero* to within the ~10^−9^ fractional accuracy of the determination.

## Data Availability

The laser-ranging data of LARES, LAGEOS and LAGEOS 2 are available at the NASA’s archive of space geodesy data CDDIS (Crustal Dynamics Data Information System)^37^.
